# A Rapid Method for Optimizing Running Temperature of Electrophoresis through Repetitive On-Chip CE Operations

**DOI:** 10.3390/ijms12074271

**Published:** 2011-07-01

**Authors:** Shohei Kaneda, Koichi Ono, Tatsuhiro Fukuba, Takahiko Nojima, Takatoki Yamamoto, Teruo Fujii

**Affiliations:** 1 LIMMS-CNRS/IIS (UMI2820), Institute of Industrial Science, University of Tokyo, Tokyo 153-8505, Japan; E-Mail: shk@iis.u-tokyo.ac.jp; 2 JST CREST, Tokyo 102-0075, Japan; 3 Enplas Corporation, Saitama 332-0034, Japan; E-Mail: k-ono@iis.u-tokyo.ac.jp; 4 Center for International Research on Micronano Mechatronics, Institute of Industrial Science, University of Tokyo, Tokyo 153-8505, Japan; 5 Ocean Alliance, University of Tokyo, Chiba 277-8564, Japan; E-Mail: bafuk@iis.u-tokyo.ac.jp; 6 College of Liberal Arts and Sciences, Kitasato University, Kanagawa 252-0373, Japan; E-Mail: nojima@kitasato-u.ac.jp; 7 Department of Mechano-Aerospace Engineering, Tokyo Institute of Technology, Tokyo 152-8550, Japan; E-Mail: yamamoto@mes.titech.ac.jp

**Keywords:** on-chip CE, denaturant electrophoresis, DNA separation, peptide nucleic acid

## Abstract

In this paper, a rapid and simple method to determine the optimal temperature conditions for denaturant electrophoresis using a temperature-controlled on-chip capillary electrophoresis (CE) device is presented. Since on-chip CE operations including sample loading, injection and separation are carried out just by switching the electric field, we can repeat consecutive run-to-run CE operations on a single on-chip CE device by programming the voltage sequences. By utilizing the high-speed separation and the repeatability of the on-chip CE, a series of electrophoretic operations with different running temperatures can be implemented. Using separations of reaction products of single-stranded DNA (ssDNA) with a peptide nucleic acid (PNA) oligomer, the effectiveness of the presented method to determine the optimal temperature conditions required to discriminate a single-base substitution (SBS) between two different ssDNAs is demonstrated. It is shown that a single run for one temperature condition can be executed within 4 min, and the optimal temperature to discriminate the SBS could be successfully found using the present method.

## 1. Introduction

Over the past two decades, microfluidic devices for high-throughput (bio)chemical analyses, so-called “micro total analysis systems” (μTAS) or “lab-on-a-chip”, have been attracting enormous attention and growing rapidly [1,2]. In particular, microfluidic devices for miniaturized capillary electrophoresis (CE) apparatus, to be referred to as “on-chip CE” or “CE chip”, have been well developed [3–5], and nowadays some of them have been commercialized as fruits of this research field [6,7]. On-chip CE devices have been applied to analyses of biomolecules including DNA [8,9], amino acids [5,10], proteins [11,12] *etc*. In particular, on-chip CE device for DNA analyses; e.g., DNA sizing [13,14], PCR product analysis [8,15], restriction fragment analysis [15] and DNA sequencing [9,16], are well sophisticated. In addition, some devices for DNA analysis were highly functionalized with pre-gel reaction components [17–19], detection components [20,21] and droplet-based liquid handling components [22,23].

In general, conventional on-chip CE devices have two advantages to achieve rapid separation. One is the method to form sample plug with a quite small volume of less than 1 nL by switching the electric field, represented by “cross-injection” method [8]. Second is the capability to apply high field strength without harmful effects by joule heating due to lower thermal mass of the device by miniaturization. Another feature of on-chip CE is that the whole operation to conduct electrophoresis (from sample loading, to plug formation and separation) is carried out just by switching the electric field. This allows us to repeat consecutive run-to-run operations on a single device with high reproducibility and reliability [8,10].

In this paper, we propose a rapid method to determine the optimal running temperature for denaturant electrophoresis to detect single-base substitution (SBS) of DNAs using peptide nucleic acid (PNA) molecule [24,25] as an oligoprobe [26,27] through repetitive on-chip CE operations by utilizing the high-speed separation and the capability of multiple runs on a single device. As an alternative to Southern hybridization, the combination of pre-gel hybridization of the PNA probe with DNA samples and following denaturant electrophoretic separation is a simple and easy technique to rapidly identify specific sequences. However, to apply this technique to SBS detection labor andtime-consuming processes to optimize the running temperature and concentration of chemical denaturant (such as urea or formamide) added to the sieving matrix are required. Thus, we designed and fabricated a temperature controlled on-chip device to validate the proposed method by determining the optimal temperature condition to allow discrimination of a SBS between two different DNAs using a PNA probe. Consequently, the shorter time required for a single run at one temperature and repetitive operations on the device allow us to rapidly determine the optimal temperature to detect the SBS.

## 2. Experimental Section

### 2.1. Design and Fabrication of Temperature Controlled On-Chip CE Device

**Figure 1:**
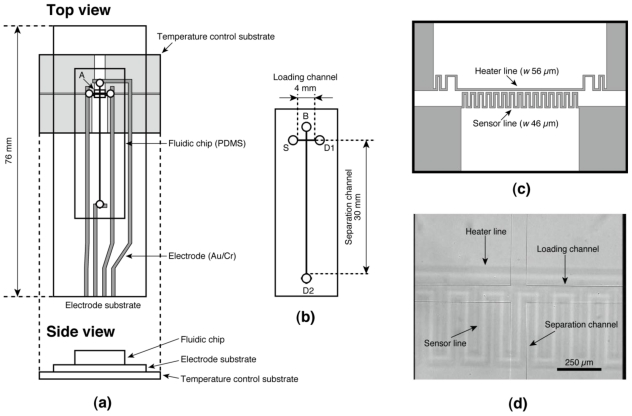
Design of the temperature controlled on-chip capillary electrophoresis (CE) device. (**a**) Top view and side view; (**b**) Magnified view of the fluidic chip. The fluidic chip has cross-shaped microchannels (90 μm width and 25 μm depth) connected to four access ports. The main working region of the temperature control substrate (region A shown in (**a**)) is detailed in (**c**); (**d**) Optical micrograph of the intersection of microchannels on the assembled device. The heater and sensor lines of the temperature control substrate are located below the loading channel on the fluidic chip.

We designed and fabricated the device illustrated in Figure 1. It consists of a fluidic chip made of poly(dimethylsiloxane) (PDMS) and two glass substrates; one is referred to as an “electrode substrate” equipped with Au/Cr electrodes for electrophoresis, and the other is the “temperature control substrate” onto which indium tin oxide (ITO) heater/sensor structures was patterned [28,29] to control the running temperature of electrophoresis (Figure 1a). The fluidic chip has cross-shaped microchannels (90 μm wide and 25 μm deep) as shown in Figure 1b. The four access ports on the fluidic chip are referred to as “source port” (S), “buffer port” (B), and “drain port” (D1 and D2) (Figure 1b). Figure 1c shows the design of the heater/sensor structures on the temperature control substrate. The fluidic chip was fabricated through the soft-lithography process described elsewhere [11,19,30]. Briefly, a negative master was fabricated on a glass substrate using ultrathick photoresist (SU-8 2025, Kayaku MicroChem, Tokyo, Japan). Prepolymer of PDMS (SYLPOT 184, Dow Corning Toray, Tokyo, Japan) was cast onto the master and cured at 75 °C for 2 h in a convection oven. After peeling off the cured PDMS from the master, it was diced to a chip size (14 mm × 42 mm × 2 mm) and access ports of around 6.3 μL volume were punched on the chip using a biopsy punch (2 mm diameter, Kai Industries, Seki, Japan). The microchannels’ width and depth are 90 and 25 μm, respectively. The electrodes on the electrode substrate were fabricated on a microscope slide glass (S91112, 52 mm × 76 mm × 1 mm, Matsunami Glass Ind., Osaka, Japan) as follows. The glass slide was cleaned by an alkaline detergent (Cica Clean LX-IV, Kanto Chemical, Tokyo, Japan) using an ultrasonic rinsing cleaner for 30 min. After rinsing with deionized (DI) water and drying, both a Cr layer of 30 nm thick and an Au layer of 50 nm thick were fabricated on the glass slide using a vacuum thermal evaporation apparatus (VCP-410A, ULVAC, Kanagawa, Japan). Then the designed electrode pattern was fabricated on the glass slide through a conventional wet etching process with a positive photoresist (AZ P1350, AZ Electronic Materials Japan, Shizuoka, Japan) as etching mask. The glass slide was diced by a cutter to be use as the electrode substrate, then the remaining photoresist was removed by a solvents rinsing (acetone, 2-propanol, and DI water) and was cleaned by soaking in piranha solution (mixture of sulfuric acid and hydrogen peroxide at a 3:1 volume ratio) for 30 min. After fabrication of the electrode substrate, the fluidic chip was reversibly bonded to the electrode substrate. To suppress the electroosmotic flow during electrophoresis due to the glass surface of the electrode substrate, the bonded chip and substrate was baked at 200 °C for 3 h for coating the glass surface by low molecular weight PDMS. The heater/sensor structures on the temperature control substrate were fabricated on a glass slide on which an ITO film was sputtered (10 Ω/sq, 100-nm thick layer, 42 mm ×102 mm × 1 mm, Toa Optical Technologies, Tokyo, Japan). Etching of the ITO film was carried out by soaking in an acid based etchant (6 M HCl and 0.2 M FeCl_3_ in DI water) for 30 min using the same positive photoresist as etching mask. After dicing the glass slide to be the size of the temperature control substrate, the remaining photoresist was removed by solvent rinsing and cleaned with oxygen plasma using a reactive ion etching machine (RIE 10NR, Samco International, Kyoto, Japan). The temperature control substrate was annealed at 200 °C for 3 h to suppress the drift of the electrical resistance of the heater/sensor structures. Finally, the temperature controlled on-chip CE device was completed by placing the bonded chip and electrode substrate on the temperature control substrate with alignment by eye (Figure 1d) and the chip was fixed by a couple of clips.

### 2.2. Process of Repetitive On-Chip CE Operations with Different Temperature Conditions

**Figure 2:**
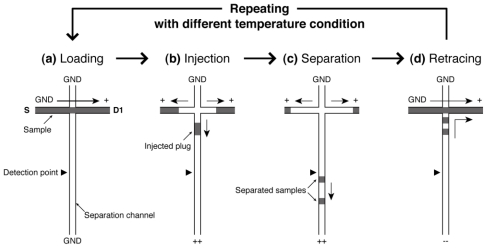
Operations of repetitive on-chip CE. (**a**) The sample analyte is introduced into the loading channel by applying voltage between S and D1 port at a certain CE temperature; (**b**) Sample plug is formed by switching the voltage and the plug is injected into the separation channel; (**c**) The injected plug is separated into different bands by their difference in mobility. The signals from the bands are detected at a certain detection point; (**d**) After detection, the separated samples are directed into the D1 port by switching the voltage again. Then, the next run is conducted under a different temperature condition. This cycle (**a**–**d**) is repeated until the optimal running temperature is found.

Figure 2 shows the process of repetitive CE operations with different temperature conditions. At first, one run of electrophoresis from sample loading through to separation is performed at a temperature condition by switching the applying voltages to the ports (Figure 2a–c). Second, the separated samples in the separation channel are directed to the drain port (D1) as shown in Figure 2d. Then the next run is started at a different temperature. This cycle is repeated until an optimal temperature condition will be found. Since the whole operation is performed only by switching the applying voltages, the repetitive on-chip CE operations can be easily and rapidly realized by programming the voltage sequence on a single device without any exchange of sieving matrix that is required in the case of a conventional CE apparatus.

### 2.3. Repetitive On-Chip CE Operations for SBS Detection

As a simple model of SBS, we selected the single-base mutation R533X on the cystic fibrosis transmembrane conductance regulator (CFTR) gene [31]. Two 15-mer single-stranded DNAs (ssDNA) purchased from Nihon Gene Research Laboratory (Miyagi, Japan) were used as wild-type and mutant samples. Sequences are as follows: wild-type (FITC-5′-AGG TCA ACG AGC AAG-3′), mutant (FITC-5′-AGG TCA ATG AGC AAG-3′). PNA is a mimic molecule of DNA and it can hybridize with DNA by following Watson-Crick base pairing. As a probe, a 15-mer PNA purchased from Fasmac (Kanagawa, Japan) with a complimentary sequence (*N*→*C* terminus; –O–O–CTT GCT CAT TGA CCT) to the mutant DNA was used. “O” stands for a six carbon spacer unit. The PNA probes were mixed with each ssDNA sample in a 0.2 mL microtube (10.0 μM PNA, 1.0 μM sample DNA with 10 mM Tris-HCl buffer), respectively. The microtubes were placed in a heating block set at 95 °C for 10 min, and then cooled to room temperature gradually. A 0.5% (w/v) hydroxyethyl cellulose (HEC) (M.W.: 90,000–105,000, Polysciences, PA, USA) polymer in TBE buffer containing 4 M urea (Nacalai Tesque, Kyoto, Japan) was used as sieving matrix. Addition of urea as chemical denaturant is required to keep the running temperature within the controllable temperature range of the device (typically less than 60 °C) [26,27]. All the aqueous solutions were prepared with deionized water produced by a Milli-Q AP system (Nihon Millipore, Tokyo, Japan). The polymer solution as sieving matrix was introduced into the microchannel by autonomous solution filling utilizing the gas permeability of PDMS [32,33]. After the filling, the polymer solution in the sample port was replaced with the sample solution of ssDNA hybridized with PNA probes. All other ports were kept filled with the same polymer solution. The voltage sequence for the repetitive on-chip CE shown in Table 1 is applied using a PC controlled high voltage power supply (HVS4448 3000 V, Labsmith, CA, USA). Fluorescence images in the microchannel were monitored using a fluorescence microscope (BX51WI, Olympus, Tokyo, Japan) and a 3CCD camera (Exwave HAD, Sony, Tokyo, Japan). The obtained images were analyzed using a laboratory made image analysis software. The temperature sensor on the temperature control substrate works as a resistive temperature detector. A digital multimeter (R6451A, Advantest, Tokyo, Japan) was used for monitoring the electrical resistance. For temperature control of the device, temperature-resistance conversion curves are obtained in advance. The relationship between the resistance and temperature is highly linear [28,29]. A DC power supply (E3631A, Hewlett Packard, CA, USA) is used to drive the heater structure on the temperature control substrate, and the running temperature on the substrate is controlled by adjusting the output of the DC power supply manually. To check the thermal profile on the temperature control substrate, an infrared thermocamera (NeoThermo TVS-600, Nippon Avionics, Tokyo, Japan) was used. A glass slide coated with high emissivity material (THI-1B, Tasco Japan, Osaka, Japan) is used for the temperature profile observation as a substitution of the electrode substrate.

## 3. Results and Discussion

The combined use of PNA probes hybridized to the region including mutation and the following electrophoretic separation is one simple and easy way to detect SBS on DNA [26]. To detect SBS, the electrophoresis should be run with an appropriate temperature condition to discriminate difference in electropherograms between wild-type and mutant DNAs. In other words, one needs to obtain the temperature conditions or the concentration of chemical denaturants required to melt the PNA/DNA hybrid with a mismatch, whereas those perfectly complementary are stable [26]. Although, studies exist on the prediction of the melting temperature of PNA/DNA hybrids [34–36], precise prediction is difficult due to the addition of chemical denaturants into the sieving matrix to keep the running temperature within the controllable temperature range of the device. Therefore, in the conventional CE apparatus, we have to find the optimal temperature or the concentration of chemical denaturants through trial and error experiments.

**Figure 3:**
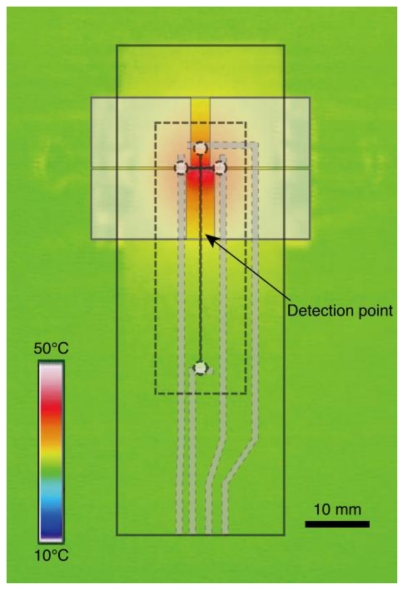
Thermograph of the glass slide mounted on the temperature control substrate. The temperature is adjusted to 42 °C. The illustrations shown by doted lines indicate the corresponding structures on the actual device.

**Figure 4:**
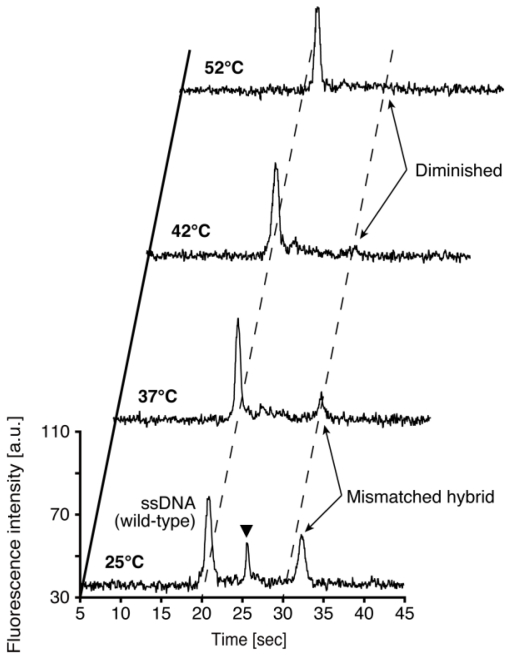
Result of consecutive on-chip CE operations with wild-type DNA hybridized with PNA probes at four different temperature conditions. Samples contained 1.0 μM wild-type DNA hybridized with 10.0 μM PNA probes. 0.5 % (w/v) HEC polymer solution containing 4 M urea is used as sieving matrix. The detection point is located 10 mm downstream from the intersection of the microchannels.

Figure 3 shows an infrared thermograph of the temperature control substrate at 42 °C. The region of the loading channel is heated with high uniformity. The temperature of the detection point located 10 mm downstream from the intersection of microchannels is lower than that of the intersection. Repetitive on-chip CE operations run at four different temperatures for the wild-type DNA hybridized with the PNA probes are demonstrated as shown in Figure 4. The sequence of the PNA probes has one base mismatch against wild-type DNA. Since the PNA molecule has neutral charge in its backbone, the mobility of wild-type DNA hybridized PNA probes denoted as “mismatched hybrid” (at 25 °C and 32 °C in Figure 4), is slower than that of the ssDNA. The peak of mismatched hybrid is diminished at both 42 °C and 52 °C. Therefore, we assumed that the temperature of the transition state suitable for the SBS detection exists between 37 °C and 42 °C. The peak between ssDNA and mismatched hybrid indicated by a solid triangle in Figure 4 at 25 °C might originate from the reannealed products of DNA/PNA hybrid after injection operation. We presume that the reannealed products are generated due to the lower temperature of detection point than that of loading channel as shown in Figure 3. The required time for one run at each temperature condition is within 4 min. Although, no significant change of the background level is observed during the consecutive four runs, the number of repetitive runs of this method is limited by the loss of sample and polymer solution due to the effect of evaporation. By adding extra sample or polymer solutions, we can increase the limit of the number of repeatable runs. However, there will be a change in the composition of solution in the ports by the effect of evaporation that induces the change in the mobility of analytes. Addition of internal standards to the sample solution would be one of the promising ways to cancel the effect.

**Figure 5:**
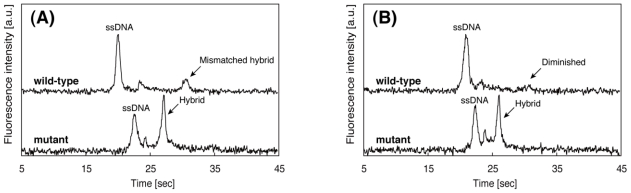
Electropherograms of wild-type and mutant DNA samples run at (**A**) 37 °C and (**B**) 42 °C. All other separation conditions are the same as in Figure 4.

Figure 5 shows resultant electropherograms of wild-type and mutant where the latter is the SBS in the target region hybridized with PNA probes, run at two different temperatures. The sequence of the PNA probes is complementary to the mutant DNA and there is one base mismatch against wild-type DNA. As shown in Figure 5A, at 37 °C, both wild-type and mutant samples display the peak corresponding to the DNA/PNA hybrids. The mobility of the mismatched hybrid is lower than that of the perfectly matched one. We assumed that this is due to the difference of hydrodynamics of radius of hybrids. The mismatched hybrid has a larger hydrodynamic radius than that of the perfectly matched hybrid. At 42 °C, the mismatched hybrid is melted thus its peak disappears, whereas the perfectly matched hybrid remains bound. This different behavior in the electropherogram allowed us to detect the SBS. The SBS detection using PNA probes could be successfully performed at 42 °C, thus we can validate the effectiveness of the repetitive on-chip CE to determine the optimal running temperature.

## 4. Conclusions

In this paper, we proposed repetitive on-chip CE as an easy and rapid method to determine optimal temperature conditions for electrophoresis. The effectiveness of the present method is validated through SBS detection using a PNA probe. Thanks to high-speed separation and the availability of consecutive run-to-run electrophoresis using on-chip CE, one run at a certain temperature condition is performed within 4 min and the optimal temperature conditions can be found using a single device without any exchange of sieving matrix that is required when using a conventional CE apparatus. The proposed method could be effectively applied to other denaturant electrophoresis for DNA variation analyses, which require the optimization of running temperatures or concentration of chemical denaturants [37,38].

## Figures and Tables

**Table 1:** Voltage sequence for repetitive on-chip capillary electrophoresis (CE).

	S (V)	D1 (V)	D2 (V)	B (V)	Time (s)
**Loading**	0	100	0	0	60
**Injection/Separation**	100	100	500	0	60
**Retracing**	0	100	−250	0	120
